# Rationale and design of the myocardial microinjury and cardiac remodeling extension study in the sodium lowering in dialysate trial (Mac-SoLID study)

**DOI:** 10.1186/1471-2369-15-120

**Published:** 2014-07-21

**Authors:** Joanna Leigh Dunlop, Alain Charles Vandal, Janak Rashme de Zoysa, Ruvin Sampath Gabriel, Lukas Mathias Gerber, Imad Adbi Haloob, Christopher John Hood, John Hamilton Irvine, Philip James Matheson, David Owen Ross McGregor, Kannaiyan Samuel Rabindranath, John Benedict William Schollum, David John Semple, Mark Roger Marshall

**Affiliations:** 1 Faculty of Medical and Health Sciences, University of Auckland, Private Bag 93311, Otahuhu, Auckland 1640, New Zealand; 2 Department of Renal Medicine, Middlemore Hospital, Counties Manukau District Health Board, Private Bag 93311, Otahuhu, Auckland 1640, New Zealand; 3 Faculty of Health and Environmental Sciences, Auckland University of Technology, North Shore Campus, Private Bag 92006, Auckland 1142, New Zealand; 4 Ko Awatea, Middlemore Hopsital, Counties Manukau District Health Board, Private Bag 93311, Otahuhu, Auckland 1640, New Zealand; 5 Renal Service, North Shore Hospital, Waitemata District Health Board, Private Bag 93503, Takapuna, Auckland 0740, New Zealand; 6 Department of Cardiology, Middlemore Hospital, Counties Manukau District Health Board, Private Bag 93311, Otahuhu, Auckland 1640, New Zealand; 7 Department of Renal Medicine, Auckland City Hospital, Auckland District Health Board, Private Bag 92024, Auckland 0740, New Zealand; 8 Department of Nephrology, Christchurch Hospital, Canterbury District Health Board, Private Bag 4710 Christchurch, New Zealand; 9 Department of Nephrology, Wellington Hospital, Wellington, New Zealand; 10 Department of Renal Medicine, Waikato Hospital, Waikato District Health Board, Private Bag 3200, Hamilton 3240, New Zealand; 11 Nephrology Service, Dunedin Hospital, Southern District Health Board, 201 Great King St, Dunedin, New Zealand; 12 Medical Affairs - Renal Asia Pacific, Baxter Healthcare Pty Ltd, P.O. Box 14062, Panmure, Auckland 1741, New Zealand

**Keywords:** Home hemodialysis, Self-care hemodialysis, Dialysis, Left ventricular mass, Left ventricular remodelling, Myocardial stunning, Sodium, Blood pressure, Intra-dialytic hypotension, Dialysate

## Abstract

**Background:**

The Sodium Lowering in Dialysate (SoLID) trial is an ongoing a multi-center, prospective, randomised, single-blind (assessor), controlled, parallel assignment clinical trial, enrolling 96 home and self-care hemodialysis (HD) patients from 7 centers in New Zealand. The trial will evaluate the hypothesis that lower dialysate [Na+] during HD results in lower left ventricular (LV) mass. Since it’s inception, observational evidence has suggested increased mortality risk with lower dialysate [Na+], possibly due to exacerbation of intra-dialytic hypotension and subsequent myocardial micro-injury. The Myocardial Micro-injury and Cardiac Remodeling Extension Study in the Sodium Lowering In Dialysate Trial (Mac-SoLID study) aims to determine whether lower dialysate [Na+] results in (i) increased levels of high-sensitivity Troponin T (hsTnT), a well-established marker of intra-dialytic myocardial micro-injury in HD populations, and (ii) increased fixed LV segmental wall motion abnormalities, a marker of recurrent myocardial stunning and micro-injury, and (iii) detrimental changes in LV geometry due to maladaptive homeostatic mechanisms.

**Methods/design:**

The SoLID trial and the Mac-SoLID study are funded by the Health Research Council of New Zealand. Key exclusion criteria: patients who dialyse > 3.5 times per week, pre-dialysis serum sodium <135 mM, and maintenance haemodiafiltration. In addition, some medical conditions, treatments or participation in other dialysis trials that contraindicate the study intervention or confound its effects, will be exclusion criteria. The intervention and control groups will receive dialysate sodium 135 mM and 140 mM respectively, for 12 months. The primary outcome measure for the Mac-SOLID study is repeated measures of [hsTnT] at 0, 3, 6, 9, and 12 months. The secondary outcomes will be assessed using cardiac magnetic resonance imaging (MRI), and comprise LV segmental wall motion abnormality scores, LV mass to volume ratio and patterns of LV remodeling at 0 and 12 months.

**Discussion:**

The Mac-SoLID study enhances and complements the SoLID trial. It tests whether potential gains in cardiovascular health (reduced LV mass) which low dialysate [Na+] is expected to deliver, are counteracted by deterioration in cardiovascular health through alternative mechanisms, namely repeated LV stunning and micro-injury.

**Trial registration:**

Australian and New Zealand Clinical Trials Registry number: ACTRN12611000975998.

## Background

The Sodium Lowering in Dialysate (SoLID) trial [1,2] is an ongoing clinical trial to evaluate the hypothesis that reducing sodium exposure in hemodialysis (HD) patients will result in lower cardiovascular (CV) risk (http://www.solid.org.nz). This hypothesis is based upon the substantial body of observational and clinical trial evidence that lowering dialysate sodium concentration ([Na+]) improves blood pressure and fluid overload, acceptance of left ventricular (LV) hypertrophy as a strong independent mortality risk [3], and an assumption lower dialysate [Na+] should ameliorate LV hypertrophy. The SoLID trial is a multi-center, prospective, randomised, controlled clinical trial, enrolling 96 patients from 7 centers. The intervention and control groups are dialysed using dialysate [Na+] 135 mM and 140 mM respectively, for 12 months. The primary outcome measure is left ventricular mass index, as measured by cardiac MRI after 12 months of treatment.

Since the design and inception of the SoLID trial, several observational studies have suggested potential harm from lower dialysate [Na+] in at-risk populations. The most robust are from the Dialysis Outcomes and Practice Patterns Study (DOPPS) [4,5]. The first of these studies reported that lower dialysate [Na+] (<137 mM) was associated with a 35% higher mortality risk compared to higher dialysate [Na+], but only in patients with lower serum [Na+]. The second study reported that lower dialysate [Na+] was also associated with higher hospitalization risk. Similar associations have been found in other studies [6]. Of note, the authors of the DOPPS papers identified those patients with serum sodium < 137 mM as having a discrete “frail” phenotype, with a higher proportion of diabetes mellitus and CV disease.

**Figure 1:**
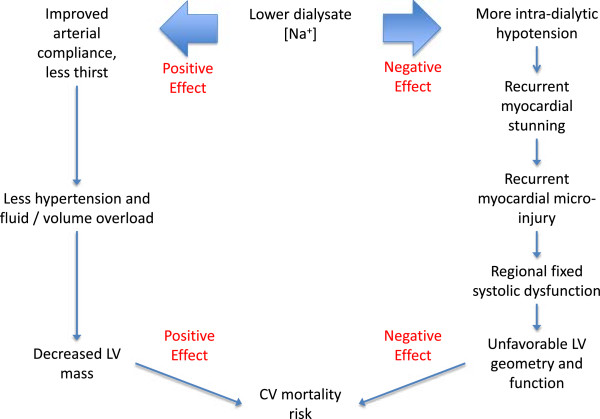
Causal diagram relating low sodium dialysate during HD to CVr mortality risk.

Due to the observational nature of the studies, any causal association between lower dialysate [Na+] and increased mortality risk is speculative. However, the studies do raise the possibility of harm, especially in frailer patients. One possible causal mechanism involves intra-dialytic hypotension (IDH), as shown in Figure 1. IDH complicates 20-30% of HD sessions, and is more likely to occur with lower dialysate [Na+] [7-9]. The reduction in myocardial perfusion pressure with IDH is compounded by profound myocardial hypoperfusion that is ubiquitous during HD, even in ostensibly ‘healthy’ dialysis patients without a cardiac history or risk factors for CV disease [10,11]. Intra-dialytic myocardial stunning is common in the myocardial regions with the greatest hypoperfusion, and manifests as regional LV dysfunction. Repeated episodes of intra-dialytic myocardial stunning lead to regions of fixed systolic dysfunction, probably as a result of underlying myocardial fibrosis [12]. It is therefore unsurprising that IDH is strongly associated with myocardial stunning and micro-injury, and all-cause patient mortality [13-16]. It is possible that any benefit from lower dialysate [Na+] conferred by reducing inter-dialytic hypertension and LV mass might be offset by increased episodes and severity of IDH and subsequent myocardial stunning and micro-injury. HD patients of the “frail” phenotype are likely to be most at risk of harm from IDH because of their reduced coronary flow reserve, and therefore to have a reduced threshold for developing myocardial ischemia in the setting of reduced coronary perfusion pressure [17-22].

This causal mechanism linking lower dialysate [Na+] to poorer outcomes is only speculative, however, and several clinical tenets argue against its validity. Firstly, lower dialysate [Na+] is associated with lower inter-dialytic weight gain (IDWG) [23-35] and consequently less fluid removal during dialysis, factors which are protective against IDH [36] . Secondly, elevation in plasma [Na+] is likely to occur during treatment with a higher dialysate [Na+]. Elevation of plasma sodium by 1-3 mM within the normal range is known to induceand “stiffening” of the vascular endothelium independently of extra-cellular fluid volume [37]. This is thought to be due to down- regulation of vasodilatory nitric oxide formation, in the presence of higher serum [Na+] [38,39]. Arterial stiffness reduces myocardial perfusion, and reduces the threshold for myocardial ischemia, especially in subendocardial regions for those with LV hypertrophy [40,41]. Arterial stiffness may also impair pressor responses to sympathetic activation during fluid removal, and reduce capacity for appropriate compensatory mechanisms during fluid removal [42]. Finally, laboratory science has demonstrated that increased sodium concentration can directly induce cellular hypertrophy; hence, higher dialysate sodium itself may be an important determinant of myocardial hypertrophy or LVH [43].

Overall, there is clinical equipoise around the use of lower dialysate [Na+] and uncertainty with respect to CV outcomes. The prime concern, however, is that there could be potential harm from the intervention through exacerbation of IDH and subsequent myocardial micro-injury. Frequency of IDH is a secondary outcome in the SoLID trial and is monitored by the trial’s Data Monitoring Committee. However, these measurements alone are unlikely to be sufficiently sensitive to detect or quantify cardiac micro-injury. The Myocardial Micro-injury and Cardiac Remodeling Extension Study in the Sodium Lowering In Dialysate Trial (Mac-SoLID study) addresses this shortcoming, and aims to determine whether lower dialysate [Na+] results in (i) increased levels of high-sensitivity Troponin T (hsTnT), a well-established marker of intra-dialytic myocardial micro-injury in HD populations [44-46], and (ii) increased fixed LV segmental wall motion abnormalities, a marker of recurrent myocardial stunning and cumulative micro-injury [12,13,46], and (iii) detrimental changes in LV geometry due to maladaptive homeostatic mechanisms [47,48].

## Methods/design

### Study aim and hypothesis

Our hypothesis is that lower dialysate [Na+] during HD is not unacceptably worse than standard care in causing myocardial micro-injury (non-inferiority). The proposed research will be conducted as an extension of the ongoing SoLID trial, a multi-center prospective, randomized, single-blind (outcomes assessor), active controlled, parallel assignment 3-year clinical trial [1,2].

#### Primary aim

The Mac-SoLID study aims to compare the effect of low dialysate [Na+] versus higher dialysate [Na+] during HD on changes in [hsTnT] over one year.

#### Secondary aims

The Mac-SoLID study aims to compare the effect of low dialysate [Na+] versus higher dialysate [Na+] during HD on

•Changes in cardiac segmental wall motion over one year.

•Changes in the LV geometry over one year.

### Study design and setting

The SOLID Trial is enrolling 96 adult participants on home and self-care HD from Waitemata, Auckland, Counties Manukau, Waikato, Capital & Coast, Canterbury, and Southern District Health Boards (DHBs). The rationale and protocol for the SoLID trial have been previously published [1,2]. There will be accrual of participants over 24 months, and a follow-up duration of 12 months. Participants will be randomly allocated to either lower dialysate [Na+] of 135 mM or higher dialysate [Na+] of 140 mM for 12 months duration, interventions that represent poles of customary practice with respect to dialysate [Na+] prescription in New Zealand.

### Ethical considerations

Ethical approval for the SoLID trial and the Mac-SoLID study has been obtained through the National (New Zealand) Multi-region Ethics Committee (IRB00004663) of the New Zealand Ministry of Health (IORG0000895), and each institutional review board within participating DHBs. (Waitemata FWA00003655, Auckland FWA00000503, Counties Manukau FWA00021560, Waikato FWA00003808, Capital & Coast FWA00002621, Canterbury FWA00004799, Southern FWA00004456).

### Target population and eligibility criteria

Eligibility criteria are: those on maintenance or self-care home HD, age greater than 17 years; clinically suitable for the study in the view of their treating nephrologist; pre-dialysis plasma [Na+] ≥ 135 mM; willing to participate and able to provide consent.

Exclusion criteria are: HD at a frequency greater than 3.5 times per week; treatment with maintenance hemofiltration; life expectancy of less than 12 months; scheduled for live donor kidney transplantation within 12 months of entry to the study; considered by the treating nephrologist to have concomitant illnesses or conditions that limit or contraindicate study procedures and follow-up (e.g. frequent intra-dialytic hypotension requiring fluid resuscitation, non-adherence); current enrolment in clinical studies involving anti-hypertensive medications, changes in HD operating parameters, or any other intervention that is likely to confound the outcome of the trial; currently using sodium profiling during haemodialysis treatments; documented infiltrative cardiomyopathies (amyloid, glycogen storage disease), hereditary cardiomyopathies (hypertrophic cardiomyopathy) or moderate to severe aortic valve disease (aortic stenosis, regurgitation).

### Recruitment of participants

The usual recruitment practice for the SoLID trial, previously published [1,2], will continue. Participant information and consent forms will be updated to include the two additional end-point measures that comprise the Mac-SoLID study. Those participants who are already enrolled or have already completed the SoLID trial will be informed of the Mac-SoLID study. They will be asked to sign an extra consent form agreeing to [hsTnT] being tested on their stored serum and an additional analysis of their cardiac MRIs by the trial cardiologists.

### Randomization

In the SoLID trial, those eligible after baseline assessment are randomly assigned to lower and higher dialysate [Na+] by a computer generated sequence in blocks of 4 participants. Randomization is stratified by a) treating center, and b) conventional (≤18 hours/week) versus extended-hour (>18 hours/week) HD.

### Blinding

Two blinded independent analysts will analyze all cardiac MRI data, and the results will be reconciled in accordance with the standard operating procedures of the group. Both analysts will be monitored weekly for drift.

Serum samples will be frozen and stored until the end of the trial when [hsTnT] analysis will be undertaken in a single batch, at a single laboratory. Individual samples will be de-identified.

### Interventions

Apart from dialysate [Na+], HD operating parameters for all participants will be managed in usual fashion according to local treatment goals. Dietary salt intake will be managed according to local clinical practice guidelines (Nutrition & Dietetics 2006; 63 (Suppl. 2): S35–S45), and monitored at baseline and 6-monthly using 3-day food diaries. Residual urinary Na + excretion will be monitored at baseline and 6-monthly using inter-dialytic urine collection.

#### Lower dialysate [Na+] HD

This group will undergo HD with dialysate [Na+] of 135 mM for a duration of one year, introduced gradually by changes of 1 mmol/week over a 4-week run-in period. BP will be optimized by changes to target weight and antihypertensive medications according to a standardized protocol.

#### Higher dialysate [Na+] HD

This group will undergo HD with dialysate [Na+] of 140 mM for a duration of one year, similarly introduced by changes of 1 mmol/week over an appropriate run-in period. BP will be optimized according to the standardized protocol above.

### Research outcomes and endpoints

#### Primary outcomes measure - high-sensitivity Troponin T

The primary end-point is time averaged [hsTnT], which will be measured at 3, 6, 9, and 12 months. [hsTnT] will be measured using the 4th generation Roche hsTnT assay (Elecsys), which will be performed on frozen serum samples which are being drawn at each time point. Samples will be drawn immediately prior to HD after a “long break”.

### Secondary outcomes measures

#### Segmental wall motion (SWM)

The first secondary end-point is SWM (score in units) at 12 months. SWM will be measured using cardiac MRI studies performed prior to HD treatments after a “long break”. All cardiac MRI studies will be performed in the following local centers according to a standardized protocol: The Centre for Advanced MRI (CAMRI) at the University of Auckland (http://www.mri.auckland.ac.nz), Valley Imaging and X-ray at Hutt Valley DHB, Midland MRI, Christchurch Hospital, and Dunedin Hospital. Assessment of LV function and wall motion will be performed on trueFISP cine imaging (6–7 short axis and 3 LV long axis with 20–30 cardiac phases depending on heart rate). Analysis of the images will be performed at a core laboratory at the Auckland MRI Research Group, University of Auckland, New Zealand. Each patient will have a four-dimensional mathematical model of the left ventricle created using guidepoint fitting. In all cases, volume, mass and wall thickness will be measured directly from the moving 3D curved surfaces, which track the motion of the endo- and epicardium. This method has been validated for global parameters such as LV mass, end-diastolic volume, end-systolic volume, stroke volume and ejection fraction using global gold standard model. Segmental wall motion analysis will be assessed according to a standard 17-segment model [49,50]. Each segment will be analyzed individually and scored on the basis of motion and systolic thickening and a wall motion score index will be derived. All data will be analyzed in duplicate by two blinded analysts.

#### Left ventricular geometry

The second secondary end-point is an assessment of LV geometry at 12 months. LV hypertrophy is the primary outcome of the SoLID trial, and well established as a powerful, independent predictor of CV morbidity and mortality among dialysis patients. Abnormal LV remodeling also carries an incremental risk independent of LV hypertrophy, particularly in studies of individuals free of cardiovascular disease (Cheng et al. Circ Cardiovasc Imaging 2009; 2:191–198) [51-55]. Cardiac remodeling can be described as a physiologic and pathologic condition that may occur after myocardial infarction, pressure or volume overload, inflammatory heart muscle disease, or idiopathic dilated cardiomyopathy. The exact cellular and molecular pathways responsible for LV remodeling are still unclear, although a key event is myocyte lengthening and stretch, stimulated expression of altered proteins and myocyte hypertrophy, leading to a further deterioration in cardiac performance and increased neurohormonal activation. In addition, a parallel process of increased activation of aldosterone and cytokines may also stimulate collagen synthesis, thus leading to fibrosis and remodeling of the extracellular matrix [47].

Three abnormal LV geometric patterns have been defined: concentric hypertrophy, eccentric hypertrophy, and concentric remodeling [55,56]. Each pattern appears to carry a different risk for CV events [57,58]. LV remodeling analysis will include an assessment of LV end-diastolic volume, LV mass, and mass to volume [M-V] ratio (unadjusted LV mass/LV end-diastolic volume ratio). Classification of LV geometry by echocardiography relies on measurement of relative wall thickness, and the M-V ratio is accepted as the cardiac MRI equivalent of relative wall thickness. The M-V ratio, however, lacks a well-defined normal reference range, and distributions that have been determined from healthy volunteers without co-existent coronary artery disease, hypertension, or other forms of congestive heart disease/fluid overload may not apply to those on dialysis. Accordingly, classification of patients into the three abnormal LV geometric patterns in this Mac-SoLID study will be based on distributions within the study population, rather than arbitrary partition values for M-V ratio from the literature.

#### Other outcome measures in the SOLID trial

*Left Ventricular mass index and volumes* (measured using cardiac MRI at 12 months), *Left Ventricular haemodynamics* (assessed by N-terminal of the prohormone brain natriuretic peptide and Urotensin II levels at 3, 6, 9, and 12 months), *extracellular fluid volume* (assessed by bioimpedence spectroscopy at 3, 6, 9, and 12 months), *blood pressure* (assessed by (a) intra-dialytic BP and (b) inter-dialytic BP measured as ambulatory recordings at 0, 6, and 12 months), *arterial stiffness* (assessed by carotid-femoral Pulse Wave Velocity at, 6, and 12 months and by radial Pulse Wave Analysis at 3, 6, 9 and 12 months, *thirst and xerostomia* (measured using validated inventories at 3, 6, 9, and 12 months), *intra-dialytic hypotension* (assessed as a summary measure from the preceding interval at 3, 6, 9, and 12 months), *inter-dialytic weight gain* (assessed as a summary measure from the preceding interval 3, 6, 9, and 12 months) *health-related quality of life* (measured by the KDQOL® and EuroQol EQ-5D questionnaires at 0 and 12 months), long-term CV mortality risk (assessed by high sensitivity C-reactive protein levels 3, 6, 9, and 12 months), *pre-dialysis serum ionic activity (γNa)* (3, 6, 9, and 12 months).

All measures corresponding to outcomes will also be recorded at baseline (0 months).

### Monitoring for adverse events

A formal Data Monitoring Committee (DMC) constituted by the New Zealand Health Research Council Data Monitoring Core Committee is monitoring the safety and conduct of the SoLID trial according to the terms of its charter. An independent study statistician and data manager generate both the open and closed Reports for the DMC, and have no connection to the clinical aspects of the trial. Safety reports are made and reviewed by the DMC on a 6 monthly basis.

The Mac-SoLID Extension Trial has the same intervention and study population, and will be therefore subject to the same monitoring.

### Analysis populations

For analysis of data, we define Intention to Treat (ITT) and Per Protocol (PP) analysis sets. The ITT set consists of all randomized participants who have at least one baseline measurement. All participants in the ITT set will be analyzed in the group to which they were allocated, even if they do not receive the allocated treatment, do not commence treatment, change dialysis modality or receive at kidney transplant, are lost to follow-up, or die thereby preserving the intention-to-treat framework. In particular, titration failures will remain within the ITT population as participants.

The PP analysis set consists of participants that fulfill criteria for the ITT set, have complete primary endpoint measurements and do not present any major protocol violations during the study. Average [hsTnT] is considered complete if at least one follow-up measurement is available. The following describes the major protocol deviations that will exclude patients from the PP population (minor deviations will not do so): eligibility violation; absence of any efficacy data, titration failure; other major violations will be identified by the DMC of the trial during the study and/or during the data review process. The list of all protocol deviations will be reviewed by the DMC who will determine the degree of the violation (i.e. major versus minor). Protocol deviations considered as minor will not lead to excluding patients from the PP population for analysis.

### Statistical analysis

For the primary (non-inferiority) outcome, primary analyses will be carried out on the PP analysis set, to promote conservative inference. For the secondary (superiority) outcomes, primary analyses will be done on the ITT set.

Non-inferiority tests will be carried out against one-sided alternatives, superiority tests against two-sided alternatives. All tests of significance of hypotheses concerning treatment effect parameters will be carried out using a significance level of 5%. All estimates will be produced as point estimates and as 95% confidence intervals. Per comparison error rate (PCER) control will be used in all analyses, with the exception of some subgroup analyses where False Discovery Rate (FDR) control will be implemented.

#### Blind review

Prior to unblinding, all outcome data will be reviewed by an independent statistician to determine whether a transformation, further transformation or alternative generalised linear model is necessary (for continuous covariates); whether efficiency-improving covariates should be included in the analyses; and whether the missing data strategy appears adequate. Normality assessment will follow usual visual assessments and normality tests. Candidate covariates for inclusion will be those with wide (>1 pooled standard deviation) separation at baseline, and those identified through experience or literature searches by the (blinded) investigative team as being potentially important to explain variability. They will be retained for adjustment if they clearly improve the model in terms of the coefficient or adjusted coefficient of determination, in the absence of knowledge regarding treatment.

#### Primary outcome – [hsTnT]

Measurements from the primary outcomes will be treated in a repeated measures format and assigned weights to maintain the time-averaged interpretation of the final results (equal weights in the case of complete data). Measurements will be logarithmically transformed based on evidence from the pilot data A weighted linear mixed model will be used to produce a treatment contrast, adjusted for baseline and any covariate identified in the blind review, appropriate to the time-averaged outcome.

#### Secondary outcome – SVM and LV geometry

For both secondary outcomes, we will undertake univariate (single outcome) endpoint analysis of the measurements at 12 months. We will use analysis of covariance (ANCOVA) to estimate the treatment effect, adjusting for baseline and potentially adjusting for covariates identified in the course of the blind review. The resulting treatment contrast will be reported as a point estimate and as a 95% confidence interval.

#### Contingency for non-normality

Equivalent analyses after a normalising data transformation will be carried out if non-normality of outcomes is evinced. The choice of transformation will be guided by the stabilisation of variance. When a transformation is applied, location estimates and confidence intervals will be transformed back to the original scale, with first-degree bias correction.

#### Missing data

Every effort will be made to minimise missing data. Missing outcome data will cause the patient/time point instance to be removed from the analysis. In the case of time-averaged endpoints, non-missing data will be appropriately reweighted. Joint modelling of missingness and the primary outcome will be carried out, for sensitivity assessment, if missingness exceeds 10% or is significantly different between the intervention arms at the 5% level.

#### Subgroup analyses

Subgroup analyses will be performed for the primary outcome and time-averaged BP (intra-dialytic, inter-dialytic, percentage maximum recommended daily dose of antihypertensives) according to the baseline severity of LV hypertrophy, baseline severity of hypertension, and baseline pre-dialysis plasma ionic [Na+] activity. Subgroup analyses will be carried on using interaction of the groups thus defined and the treatment arms.

### Power calculations

The statistical power calculations for the primary outcome are based on the non-inferiority hypothesis that lower dialysate [Na+] HD sodium is does not induce markedly higher [hsTnT] levels than the higher dialysate [Na+].

Published literature suggests different [hsTnT] criteria as markers for important and detectable myocardial micro-injury in this population [44,46]. For sample size estimation purposes, we retained a baseline-proportional increase of [hsTnT], and an increase of the probability of achieving [hsTnT] > 60 ng/L. In the former case, doubling of the baseline has been identified as a clinically meaningful difference. We use a relative maximal increase of 50% to define non-inferiority. In the latter case we set a relative increase of 1/3 as the threshold for inferiority.

We determined a target sample size in consideration of these two criteria, to achieve a power of 80% to declare non-inferiority, viewed as a one-sided alternative at a 5% significance level [59]. For sample size considerations, we used published literature employing the 4th generation Roche hsTnT assay (Elecsys) [44]. Baseline [hsTnT] were obtained from a NZ study of HD patients using the same assay [60].Accordingly, we assumed the mean and within-group standard deviation for [hsTnT] to be 118 and 291 ng/L respectively, with an overall correlation between baseline and follow-up value at 3 months of 0.268. We assumed the proportion of pre-dialysis [hsTnT] >60 ng/L to be 0.52 at baseline. For both the [hsTnT] value and [hsTnT] thresholding approaches, we calculated sample size using Monte Carlo simulation and mixed effects modelling adjusting for baseline value (on the log scale). Based on the original assumptions in the SOLID Trial, we allowed 25% for drop-outs.

Using a margin of non-inferiority defined by an individual relative average increase of baseline [hsTnT] of 50% or more requires a target of 33 participants per arm to achieve 80% power. This corresponds to the planned primary analysis. Using a margin defined by a relative increase of 1/3 in the probability of a participant having time-averaged [hsTnT] superior to 60 ng/L (corresponding to an increase of 17 percentage points) yields a target of 31 participants per arm to recruit to achieve the same power.

## Discussion

The SoLID trial is the first randomized controlled trial to investigate the effect of lower dialysate [Na+] upon LV structure and function, and is now close to completion. The outcomes of this research will provide definitive evidence about the efficacy of lower dialysate [Na+] to improve CV outcomes in HD populations. The SoLID trial will also provide data on important patient-centered outcomes, such as the effect of lower dialysate [Na+] on thirst, xerostomia, HRQoL and intra-dialytic hypotension. If the benefit of lower dialysate [Na+] is confirmed, other benefits might also flow on from reduced CV morbidity and mortality, including improvements in lower health resource consumption and less societal burden of treatment. Operationally, lower dialysate [Na+] is a simple and cost-free intervention, and for appropriate dialysis populations it can easily be implemented on a large-scale.

The Mac-SoLID trial enhances and complements the SoLID trial. It will provide reassurance that the potential gains in cardiovascular health (reduced LV mass), which lower dialysate [Na+] is expected to deliver, are not off-set by deterioration in cardiovascular health through alternative mechanisms, namely repeated LV stunning and micro-injury. The information forthcoming from the Mac-SoLID trial may also help us to better pinpoint those patients who fit the “frail” phenotype and are inherently unsuitable for dialysis treatment with lower dialysate [Na+].

## Abbreviations

HD: Hemodialysis; ESKD: End-stage kidney disease; LV: Left ventricular; CV: Cardiovascular; hsTnT: high-sensitivity Troponin T; CAMRI: Centre for advanced magnetic resonance imaging; DMC: Data Monitoring Committee; Mac-SoLID: Myocardial micro-injury and cardiac remodeling extension study in the sodium lowering in dialysate trial; MRI: Magnetic resonance imaging; ϒNa: Ionic sodium; SoLID trial: Sodium lowering in dialysate trial; ANCOVA: Analysis of co-variance; ITT: Intention to treat; PP: Per protocol; PCER: Per comparison error rate; GEE: Generalised estimating equation; FDR: False discovery rate.

## Competing interests

MRM is an employee of Baxter Healthcare Ltd. The other authors have no competing interests to declare.

## Authors’ contributions

MRM conceived and developed the trial, and participated in the statistical plan and helped to draft the manuscript. JLD participated in the trial design, and drafted the manuscript. ACV participated in the trial design, developed the statistical plan, and helped to draft the manuscript. RSG, IAH, and DORM participated in design and development of the trial, and helped to draft the manuscript. JRdZ and PJM participated in the trial design and coordination, and helped to draft the manuscript. CJH, LMG, JHI KSR, JBWS, and DJS participated in the trial coordination and implementation, and helped to draft the manuscript. All authors read and approved the final manuscript.

## Pre-publication history

The pre-publication history for this paper can be accessed here:

http://www.biomedcentral.com/1471-2369/15/120/prepub
